# Perceived Stress as a Predictor of Partnership Relation Quality in Polish Mothers of Preterm-Born Children

**DOI:** 10.3390/ijerph16030355

**Published:** 2019-01-27

**Authors:** Mariola Bidzan, Karolina Lutkiewicz

**Affiliations:** 1 Institute of Psychology, University of Gdansk, Bażyńskiego 4, 80-309 Gdansk, Poland; 2 The Toronto Institute for Relational Psychotherapy, Toronto, ON M4T 1K2, Canada; karolina.koz@gmail.com

**Keywords:** perceived stress, relationship quality, pre-term birth, neonatal period, early childhood

## Abstract

The aim of the study was to identify whether perceived stress and significant life changes are related with partnership relation quality in mothers of preterm-born children. The study group consisted of 260 women, who gave a preterm birth. In most cases the delivery took place in the 34–36th week of pregnancy. The research consisted of two phases. Phase 1 was carried out in the Department of Obstetrics at the Medical University of Gdansk in the neonatal period (2–3 days after birth). Phase 2 was carried out in the place of residence of the mother and child during early childhood (24–30th month of the child’s life). The following research tools were used in the first phase of the research project: Analysis of nursing and medical reports, an interview questionnaire and psychological interview, The Perceived Stress Questionnaire (PSQ) (Lavenstein, the Polish version, after Plopa, 2008), The Recent Life Changes Questionnaire (RLCQ) (Rahe and Holmes, 1975), the Polish version, after Terelak 1995), and the Dyadic Adjustment Scale (DAS) (Spanier, 1976, the Polish version, after Cieślak, 1989). In the second phase of the study the respondents were once again examined using the DAS. It was shown that stress is related to partnership relationship quality and of all its components, except cohesion. The findings demonstrated that important life events are associated with a couple’s emotional expression in the neonatal period. Stressful life events do not correlate with relationship quality.

## 1. Introduction

Preterm birth is considered a major challenge of modern perinatology. The World Health Organization (WHO) estimates that around 15 million children worldwide are born prematurely each year, and the number of preterm births is increasing steadily [1]. In Poland, the pre-term birth rate is estimated to be around 6.3–8% and is on a downward trend, but still quite high compared to other developed countries in Europe [2,3,4,5,6]. In the case of high-risk pregnancies as well as problems related to procreation, relationship quality becomes extremely significant [2,6,7,8,9] as it may affect a child’s development [10,11]. The experience of preterm labor can alter the way a couple and the whole family function during the first years of a child’s life [12], especially when prematurity is associated with developmental disorders and/or disability [4,13,14]. The postnatal period is a critical phase in the lives of mothers and newborn babies [15,16].

Stress negatively impacts the quality of a relationship [17,18,19,20,21]. It may evoke negative emotions, disturb communication between partners [22] and influence the general mental and physical well-being of each partner [17,23]. Premature birth can be an extremely stressful and traumatic experience for parents as they face numerous challenges that follow the birth of a premature infant [24]. Distress may be caused by the infant’s medical condition, survival and developmental prognosis [25]. Parental distress also may be experienced after the medical crisis has passed and the infant leaves the hospital. Attempts to care for a biologically fragile infant whose behavioral cues are frequently difficult to interpret may still evoke emotional reactions [26]. Much of the research shows that stressful life events may have disruptive effects on the functioning of a relationship [27,28,29]. Bradbury and Karney’s [30] meta-analysis of the large range of literature on marriage suggested that after a stressful event (e.g., major life events, stressful circumstances, normative transitions) the experience of distress might emerge. Other research demonstrated that stressful life events reduced husbands’ and wives’ marital satisfaction [31]. Lawrence and others [32] differentiated two groups of factors that predict marital/relationship quality in the perinatal period. The authors included stress and stressful life events into a general group of predictors of marital satisfaction. Edwards, Gibbons and Gray [33] demonstrated that an impaired couple relationship in preterm mothers was associated with a high level of parental stress. Other research also demonstrated a negative correlation between stress and marital quality [34].

A traumatic birth experience and neonatal stress not only influence partners’ relationships, but also parent–infant bonds and the general attitude towards newborn children, which in turn may affect a child’s development [11,35]. The marital relationship is an important aspect of family functioning, mainly as it contributes to the individual psychological well-being of parents and children [36]. While parental functioning after pre-term birth has been extensively examined [37], not many studies have investigated if a mothers’ stress after preterm birth is related to marital quality and satisfaction. The aim of the study was to identify whether perceived stress in mothers and significant life changes predict the quality of a relationship in mothers of preterm-born children.

## 2. Research Hypotheses

### 2.1. Neonatal Period

**Hypothesis** **1.1.** *Stress experienced by mothers of preterm-born children is related to the quality of the partnership relation in the neonatal period*.

**Hypothesis** **1.2.** *Significant changes/life events are connected to the quality of the partnership relation in mothers of preterm-born children in the neonatal period*.

### 2.2. Early Childhood Period

**Hypothesis** **2.** *Life events/stressful changes in the neonatal period are related to the quality of the partnership relation in mothers of preterm-born children in the early childhood period*.

## 3. Research Materials and Methods

This study presents part of findings from a wider research project regarding prematurely born children in collaboration with the Department of Obstetrics at the Medical University of Gdansk (under the supervision of professor Krzysztof Preis and professor Małgorzata Świątkowska-Freund) and the Institute of Psychology at the University of Gdansk (under the supervision of professor Mariola Bidzan). The research consisted of two phases. Phase 1: Department of Obstetrics at the Medical University of Gdansk, neonatal period (2–3 days after birth), establishing contact with a patient; Phase 2: the place of residence of the mother and child, the period of early childhood (24–30th month of the child’s life).

The study group included 260 women who were mothers of preterm-born children. The youngest respondent was 16 and the eldest was 45 years old. The arithmetic mean of age equaled 30.0 years (SD = 5.22 years). The majority of the examined women (79.7%) were married, while 19.3% of women were in a partnership relation. Most of the examined women had completed post-secondary education (60.3%), one in four women had completed secondary education (25.2%), 11.7% had completed vocational education and 2.8% had completed basic education. Most of the women were primigravidae (63.3%). A total of 75.4% of the women were in single pregnancy. Most of the respondents (84.1%) had no pregnancy complications. In most cases, the termination of pregnancy occurred during the 34, 35 or 36th week (64.4%). In other cases, termination occurred between 24 and 33 weeks. The median was 34.0 weeks.

All data was collected by means of:

1. Analysis of medical and nursing documentation
2. Clinical interview and psychological interview
3. Interview questionnaire
4. The following research methods:

Phase 1:

The Dyadic Adjustment Scale (DAS) (Spanier, 1976, Polish adaptation by Cieślak, 1989) [38,39]; the Perceived Stress Questionnaire (PSQ) (Lavenstein, Polish version, after Plopa, 2008) [40]; and the Recent Life Changes Questionnaire (RLCQ) by Holmes and Rahe (reviewed by Terelak, 1995) [41].

Phase 2:

The DAS (Spanier, 1976, Polish adaptation by Cieślak, 1989) [38,39].

The DAS is a 32-item self-report used to assess subjectively the quality of marital and partnership relations [42]. It allows for a comprehensive assessment of the relationship quality. The scale is divided into 4 subscales and includes the following components of marital adjustment: (a) dyadic consensus—degree to which the partners agree with each other on matters significant for their relationship e.g., friends, household tasks, religion, free time, (b) dyadic cohesion—degree of closeness and frequency partners share everyday activities together, (c) dyadic satisfaction—degree to which the partners feel generally satisfied with their relationship and hence still want to be together, (d) affectional expression—degree to which the partners express feelings to each other, affection and sex [39]. The overall result of this questionnaire is expressed as the sum of all results in the above categories. The 32 items are summed to create a total score ranging from 0 to 151. Higher results suggest a higher level of relationship quality, while lower results indicate a lower level of relationship quality. The Cronbach’s alpha reliability coefficient for the overall score in the test equaled 0.96, while the score for each subscale was 0.73 to 0.94 [33].

The Perceived Stress Questionnaire (PSQ) [40,43] assesses the general level of perceived stress and its dimensions: the sense of fatigue, irritability, worrying, mental tension, lack of joy of life, sense of pressure and sense of being overwhelmed. The instrument might be useful in clinical and health promotion work [44]. The questionnaire consists of 30 statements that refer to the period of the last 4 weeks. The PSQ is used in clinical research with a particular emphasis on its predictive validity regarding the development of stress-related disorders [43]. Severe perceived stress is expressed as the sum of all scores in the above categories. The higher the score, the higher the severe perceived stress. The Cronbach’s alpha reliability coefficient equaled >0.9 [43].

The Recent Life Changes Questionnaire (RLCQ) is a tool that assesses the frequency of occurrence of life events/changes in an individual’s life which lead to multifaceted changes and require the use of an individual’s psychological resources in order to adapt to the new conditions [45]. Both positive and negative life events can have a negative emotional undercurrent and cause stress. The RLCQ for pregnant women consists of 38 statements concerning life events. The events are considered in the following time intervals:

→ 19–24 months before pregnancy;
→ 13–18 months before pregnancy;
→ 7–12 months before pregnancy;
→ 0–6 months before pregnancy;
→ during pregnancy.

The respondent is asked to present two types of information: mark the occurrence of a stressful event in the appropriate time interval and evaluate the significance of the event in terms of how difficult it is to adapt to it. The Pearson’s correlation coefficients between the test and retest scores were 0.85 for the index based on individual quantification (*p* < 0.001) and 0.81 for the index based on normative quantification (*p* < 0.001). This determined that the reliability of the RLCQ could be considered as satisfactory [45].

## 4. Results

The analyses were performed using the SPSS (IBM, Version 23.0. IBM Corp., Armonk, NY, USA). Descriptive statistics were calculated, namely percentages, averages, standard deviations, minimum and maximum values, as well as Pearson correlation coefficients for quantitative variables. First, correlations were calculated among all variables investigated to examine initial associations. Second, hierarchical regression analyses were performed in which the quality of the relationship was explained.

### 4.1. Neonatal Period

The correlation analysis showed that the variables were inter-correlated. Hypothesis 1.1 was partially confirmed. The results indicate that the perceived stress and all of its dimensions (the sense of fatigue, irritability, worrying, mental tension, lack of joy of life, sense of pressure, sense of being overwhelmed) negatively correlate with consensus, emotional expression and satisfaction with the relationship, resulting in a lower partnership relation quality as assessed subjectively by the mother. One dimension of the relationship quality, cohesion, correlated negatively with one dimension of perceived stress, the lack of joy of life. Overall, perceived stress was not related to cohesion (Table 1). Mothers’ perceived stress was mildly associated with their partnership relation in the neonatal period.

A hierarchical regression analysis was conducted to examine whether perceived stress is associated with the partnership relation quality (Table 2). The results, including components of the relation quality, suggest that high perceived stress significantly decreases the level of affectional expression, consensus and satisfaction in a relationship.

Hypothesis 1.2 was not confirmed. The correlation analysis results indicated that there were no significant correlations between life events and partnership relation quality. Life events were not related to the partnership relation quality of mothers of preterm-born children. The results are presented in Table 3.

To examine the relative contribution of significant life events and partnership relation quality, a hierarchical regression analysis was conducted. The results of the regression analysis show that significant life events were not associated with the overall partnership relation quality. In addition, regression analyses concerning the relationship quality components suggest that life events negatively related to the level of affectional expression in a relationship. Life events were associated with the level of affectional expression in a relationship, as seen in Table 4.

### 4.2. Early Childhood Period

Correlation analysis was conducted for the relation between psychological variables in the neonatal period and the partnership relation quality during the early childhood period.

Hypothesis 2 was partially confirmed, because the conducted correlation analysis showed that the affectional expression in the partnership relation during Phase 2 of the study correlated negatively with life events (Table 5). There were no other significant correlations.

## 5. Discussion

The experience of preterm labor is a special life event for parents and is related to a high level of stress [24] due to the child’s health condition, potential medical complications and long-term developmental consequences [25,46]. Studies show that stress can have a negative impact on a couple’s relationship [17,18,19,20,21]. The findings from this study demonstrate that perceived stress in mothers of preterm-born children is negatively associated with the level of partnership relation quality of these mothers in the neonatal period. The perceived stress and all of its dimensions (the sense of fatigue, irritability, worrying, mental tension, lack of joy of life, sense of pressure, sense of being overwhelmed) are related to dyadic consensus, affectional expression and satisfaction within the relationship. Other research has also confirmed the negative correlation between stress and marital quality [29,33,34,47]. In the present study, perceived stress was related to the partnership relation quality and all of its components, except cohesion. Similar results were obtained by Lawrence et al. [32], who included stress and stressful life events into the main group of predictors of marital satisfaction. The findings are consistent with a wide body of literature on the detrimental effects on marital quality and overall satisfaction within a relationship [17,18,19,20,21]. However, the findings obtained by Graham and Conoley [34] demonstrated that stress does not predict marital quality. The study results indicate that perceived stress does not correlate with cohesion in the relationship. It can be concluded that regardless of the experience of stress, the closeness and the frequency of shared activities remained unchanged. Mothers of preterm-born children engage to the same extent in the couple’s everyday life and their common interests remain unchanged. On the other hand, dimensions such as affectional expression, overall satisfaction within the relationship and consensus were significantly connected with stress. In considering an explanation for these findings, perhaps perceived stress in mothers is impacting the quality of their relationships only because of the presence of stress. Camisasca et al. [48] suggested that for mothers, consensus and cohesion are the most important aspects of a couple’s relationship, because they are greatly connected with the parent–child relationship when it comes to having the same philosophy of family life and cooperating with partners while managing everyday life.

Much of the research shows that the occurrence of stressful events may have disruptive effects on the functioning of a relationship [27,29,47] and reduce husbands’ and wives’ marital satisfaction [28]. The present study did not confirm these findings, as the correlation analysis results indicated that significant life events were not related to the partnership relation quality of the mothers of preterm-born children. The origin of stress, whether it springs from the relation between couples or comes from elsewhere, seems significant due to the possible different effects on the relationship. Moreover, it was demonstrated that significant life changes are connected with the affectional expression in the relationship in the neonatal period. According to research by Velotti et al. [49], a decline in affectional expression towards a partner might be particularly related to stress due to the arrival of a child. Some research explained a decline of affection and sexual interaction as a natural consequence of the transition from the first year of marriage to a more mature relationship [50]. Furthermore, in this study there is a positive correlation between significant life events in the neonatal period and affectional expression in the early childhood period. It can be claimed that the more significant life changes/events in a mother’s life in the neonatal period, the lower the levels of demonstration of affection to the partner and sexual interactions are during the early childhood period. Findings obtained by Treyvaud et al. [37] demonstrate that a very preterm birth has a negative influence on parents and family functioning at 7 years after birth, which for some families is consistent with their functioning at 2 years after birth.

Our results raise important practical implications for clinical interventions for couples with prematurely born children. Studies show that a poor marital relationship increases the risk of poor developmental outcomes for children [51,52]. Couple functioning is very important, particularly in the experience of premature birth, when parents face concerns over an infant’s medical condition, survival and developmental prognosis [25]. Spousal relationships are very influential during experiences of high stress, as they can prevent stress or reduce negative reactions [53]. The partner’s resources for adapting to stressful conditions, such as mutual support capacity, can mediate between stress and the marital quality [27]. The research shows that relationship quality is particularly significant in predicting well-being and health outcomes [54], e.g., the risk of maternal infectious disease in pregnancy [55]. The research shows that marital satisfaction also affects the parent–child relationship, e.g., parental investment, responsibility, attunement or effectiveness [56,57]. Different intervention programs based on this theory may begin as early as during hospitalization, therefore potentially improving mother–infant interactions and parenting stress in the early postnatal years [53]. Expanding parental knowledge about prematurity and decreasing the level of parental stress among couples should be a priority after preterm birth. Other findings also highlight the importance for pregnant women to avoid exposure to stressful situations and educating pregnant women about stress reduction [58,59].

### 5.1. Limitations

In this prospective study, only the mother’s perceived stress was taken under consideration. Definitely an important limitation was the lack of male parent participation to see how a mother’s and father’s stress influence the relationship quality. As the present study presents findings from part of a wider project, socio-demographic data were not taken into consideration. However, many of the findings on the effects of prematurity on family relationships were modified for example, by socio-economic status and parents’ education [60]. Prematurity may have many different forms, as it depends on pregnancy termination. In terms of the representation of our population, the pregnancy termination restriction could appear as a limitation to our study. This is because more than half of the women gave birth at 34, 35 or 36 weeks, which is considered late prematurity. It would be interesting to take into consideration perceived stress among mothers with infants with very low birth weight, or extremely premature infants who are exposed to more invasive treatments associated with all morbidities [61]. Another limitation of the study is the use of self-reported data. Using self-reports together with interviews, where partners could describe their relationship and stressful circumstances, would enrich the results. The research does not present causality between the measured variables, which limits the interpretation of the results.

### 5.2. Future Directions

Research by Spinelli et al. [62] has shown that mothers of preterm-born children do not form a homogeneous group. In the future it would be important to take into account individual differences in the analysis of parental stress, which may depend on the characteristics of the child and the mother’s experience, including education. It would be beneficial to investigate the sources of a mother’s stress in order to provide appropriate support to parents of preterm-born children [4,63].

## 6. Conclusions

The aim of this study was to highlight the importance of expanding interventions and support for couples to overcome the stress of premature labor. This study focuses on the nature of spouse/partner relationship quality and maternal stress associated with preterm birth. The findings emphasize the associations between stress and stressful life events on a couple’s functioning.

## Figures and Tables

**Table 1:** Pearson’s linear correlation coefficient for general relationship satisfaction and its components with a sense of stress and its components.

Variable	*M* (*SD*)	PS	SE	I	W	MT	L	FP	O
CON	52.78 (7.24)	0.34 **	0.28 **	0.33 **	0.32 **	0.38 **	0.39 **	0.28 **	−0.28 **
COH	19.53 (3.68)	−0.04	−0.002	−0.12	−0.01	−0.07	0.18 **	−0.03	−0.05
SAT	41.32 (5.25)	0.33 **	0.27 **	0.39 **	0.31 **	0.35 **	0.36 **	0.29 **	−0.26 **
AE	10.42 (1.74)	0.27 **	0.27 **	0.31 **	0.23 **	0.33 **	0.31 **	0.30 **	−0.26 **
GSAT	124.05 (15.09)	0.32 **	0.26 **	0.35 **	0.29 **	0.36 **	0.39 **	0.27 **	−0.26 **

Annotation. M—Median; CON—Consensus in a relationship; COH—Cohesion in a relationship; Sat—Satisfaction in a relationship; AE—Affectional expression; GSAT—General satisfaction from the relationship; PS—Perceived stress; SE—Sense of exhaustion; I—Irritability; W—Worrying; MT—Mental tension, L—Lack of joy of life; FP—Feeling of pressure; O—Overload; ** *p* < 0.01.

**Table 2:** Hierarchical regression analysis in which the quality of the partnership and its components were explained variables.

	CON		COH		SAT		AE		GSAT	
	*β*	Δ*R*^2^	*β*	Δ*R*^2^	*β*	Δ*R*^2^	*β*	Δ*R*^2^	*β*	Δ*R*^2^
PS	−0.28 **		−0.04		0.28 **		−0.25 **		−0.26 **	
LE Total ***R*^2^**	−0.07	0.563 **	−0.05	0.312 **	−0.10	0.487 **	−0.17 *	0.336 **	−0.10	0.595 **

Annotation. CON—Consensus in a relationship; COH—Cohesion in a relationship; SAT—Satisfaction in a relationship; AE—Affectional expression; GSAT—General satisfaction from the relationship; PS—Perceived stress, LE—Life events/changes; * *p* < 0.05; ** *p* < 0.01. *β*—Standardized Beta Coefficient, Δ*R*^2^—the squared semi-partial correlation.

**Table 3:** Correlation coefficients for the relationship of overall satisfaction with the relationship and its components to life events.

Variable	*M* (*SD*)	LE ^a^
CON	52.78 (7.24)	−0.05
COH	19.53 (3.68)	0.03
SAT	41.32 (5.25)	−0.06
AE	10.42 (1.74)	−0.10
GSAT	124.05 (15.09)	−0.05

Annotation. CON—Consensus in a relationship; COH—Cohesion in a relationship; SAT—Satisfaction in a relationship; AE—Affectional expression; GSAT—General satisfaction from the relationship, LE—Life events/changes; ^a^ Pearson’s linear correlation coefficients.

**Table 4:** Hierarchical regression analysis in which the quality of the partnership and its components were the explained variables.

	CON		COH		SAT		AE		GSAT	
	*β*	Δ*R*^2^	*β*	Δ*R*^2^	*β*	Δ*R*^2^	*β*	Δ*R*^2^	*β*	Δ*R*^2^
LE Total ***R*^2^**	−0.07	0.563 **	−0.05	0.312 **	−0.10	0.487 **	−0.17 *	0.336 **	−0.10	0.595 **

Annotation. CON—Consensus in a relationship; COH—Cohesion in a relationship; SAT—Satisfaction in a relationship; AE—Affectional expression; GSAT—General satisfaction from the relationship; LE—Life events/changes; * *p* < 0.05; ** *p* < 0.01.

**Table 5:** Correlation coefficients for the general relationship of satisfaction with the relationship and its components to life events in the second round of the study.

Variable	*M* (*SD*)	LE ^a^
CON 2	49.73 (7.69)	−0.24
COH 2	18.11 (5.14)	−0.31
SAT 2	36.54 (5.91)	−0.26
AE 2	9.14 (1.83)	−0.40 *
GSAT 2	113.51 (17.64)	−0.32

Annotation. CON 2—Consensus in a relationship (in the second round of the study); COH 2—Cohesion in a relationship (in the second round of the study); SAT 2—Satisfaction in a relationship (in the second round of the study), AE 2—Affectional expression (in the second round of the study); GSAT 2—General satisfaction from the relationship (in the second round of the study), LE—Life events/changes (in the second round of the study). ^a^ Pearson’s linear correlation coefficients; * *p* < 0.05.
